# Pregnancy outcomes after implementation of an induction of labor care pathway

**DOI:** 10.1016/j.xagr.2023.100292

**Published:** 2023-11-18

**Authors:** Monica A. Lutgendorf, Megan Northup, Jeffrey Budge, Marie Snipes, Jamie Overbey, Anne Taylor, Amanda Simsiman

**Affiliations:** 1 Department of Gynecologic Surgery and Obstetrics, Uniformed Services University of the Health Sciences, Bethesda, MD (Dr Lutgendorf); 2 Department of Gynecologic Surgery and Obstetrics, Naval Medical Center San Diego, San Diego, CA (Drs Northup and Simsiman); 3 Office of Clinical Quality Management, Naval Medical Center San Diego, San Diego, CA (Mr Budge); 4 Department of Mathematics and Statistics, Kenyon College, Gambier, OH (Dr Snipes); 5 Department of Pediatrics, Naval Medical Center San Diego, San Diego, CA (Dr Overbey); 6 Mother Infant Nursing Department, Naval Medical Center San Diego, San Diego, CA (Ms Taylor).

**Keywords:** clinical care pathway, clinical outcomes, clinical standardization, induction of labor, process improvement

## Abstract

**BACKGROUND:**

Induction of labor is common; however, the optimum clinical strategy for induction of labor is less clear. Variations in clinical practices related to induction of labor may lead to increased complications and longer induction of labor times.

**OBJECTIVE:**

This study aimed to analyze whether the implementation of an evidence-based standardized care pathway improves the clinical outcomes associated with induction of labor.

**STUDY DESIGN:**

This was an approved quality improvement project implementing a clinical care pathway for induction of labor. Moreover, this was a retrospective cohort study of inductions of labor for 5 months before (January 2018 to May 2018) and 14 months after (August 2018 to September 2019) the implementation of the care pathway. The primary outcome was time from admission to delivery. Time from admission to delivery was stratified by mode of delivery. The secondary outcomes included chorioamnionitis, endometritis, neonatal intensive care unit admissions, cesarean delivery, postpartum hemorrhage, and a composite of unanticipated outcomes (chorioamnionitis, endometritis, neonatal intensive care unit admissions, cesarean delivery, and postpartum hemorrhage). In addition, pathway adherence was analyzed. The outcomes were analyzed using 2-tailed *t* tests for continuous data and the Fisher exact test and chi-square tests for categorical data. Propensity score matching was used to assess for confounding by potential covariates.

**RESULTS:**

A total of 1471 inductions of labor were reviewed, with 392 inductions of labor before the implementation of the care pathway and 1079 inductions of labor after the implementation of the care pathway. The pathway was associated with a nonsignificant reduction in the time from admission to delivery by 1.2 hours (from 23.4 to 22.2 hours; *P*=.08). There was a nonsignificant increase in the time to cesarean delivery before (28.2 hours) and after (28.8 hours) protocol implementation (*P*=.71). There was a significant decrease in the time to delivery by 1.7 hours for vaginal deliveries (from 22.2 to 20.5 hours) after protocol implementation (*P*=.02). There was a significant decrease in chorioamnionitis (from 12.5% to 6.0%; odds ratio, 0.44; 95% confidence interval, 0.29–0.67), a significant decrease in endometritis (from 6.9% to 2.6%; odds ratio, 0.36; 95% confidence interval, 0.20–0.65), and a significant decrease in composite unanticipated outcomes (from 56.9% to 36.6%; odds ratio, 0.46; 95% confidence interval, 0.34–0.56) after the implementation of the care pathway. There was no significant difference in postpartum hemorrhage (from 7.9% to 6.1%; odds ratio, 0.76; 95% confidence interval, 0.48–1.22), neonatal intensive care unit admissions (from 18.1% to 14.0%; odds ratio, 0.74; 95% confidence interval, 0.54–1.02), or cesarean deliveries (from 19.6% to 20.1%; odds ratio, 1.03; 95% confidence interval, 0.76–1.40) after the implementation of the care pathway. Pathway adherence varied, ranging from 50% to 89%.

**CONCLUSION:**

The introduction of a standardized induction of labor pathway was associated with a nonsignificant reduction in the time from admission to delivery by 1.2 hours and improved pregnancy outcomes, including decreased infections and unanticipated outcomes. Further opportunities for improvements in clinical outcomes may be realized with increased compliance with the care pathway.

**AJOG Global Reports at a Glance**

**Why was this study conducted?**

This study aimed to determine the effect of a standardized induction of labor (IOL) protocol on the time to delivery and outcomes related to IOL.

**Key findings**

This study found that implementation of a standardized IOL pathway was associated with a nonsignificant decrease in the mean IOL time by 1.2 hours and with significant improvements in pregnancy outcomes, including a decrease in infections and composite unanticipated outcomes (infection, neonatal intensive care unit admission, cesarean delivery and postpartum hemorrhage).

**What does this add to what is known?**

This study emphasizes the importance of standardized clinical care and decreased clinical variation related to IOL.

## Introduction

Induction of labor (IOL) in the United States has increased over time and is currently performed in approximately 30% of all deliveries.^1^ IOL is indicated for both maternal and fetal indications when the risks of continuing the pregnancy outweigh the risks of IOL and delivery. Recent data regarding the benefits of IOL in nulliparous women at 39 weeks of gestation^2^^,^^3^ and hypertension guidelines^4^^,^^5^ have led to increased IOLs. At our institution, the IOL rate is approximately 40% with significant variation in practices among clinicians.

Although general recommendations exist, including time for cervical ripening, cervical dilation, and the second stage of labor,^6^^,^^7^ previous studies have been limited by small sample sizes,^8^ and clinicians must choose from many options, including pharmacologic and mechanical methods. There is evidence that clinical standardization of IOL leads to superior patient outcomes.^9^ Previous studies have shown that standardized labor management results in a decreased incidence of women in latent labor at 12 hours, eliminates failed IOL as an indication for cesarean delivery (CD) in multiparous women,^10^ lowers the rates of failed IOLs, shortens the time in labor,^11^ and decreases the time from initiation of IOL to delivery, with no difference in outcomes, including postpartum hemorrhage (PPH), chorioamnionitis, neonatal intensive care unit (NICU) admission, or CDs.^12^

Clinical pathways are evidence-based guidelines for the management of patients that include essential steps and local protocols used by multidisciplinary teams to decrease clinical variation and improve outcomes.^13^ Clinical pathways include 5 necessary criteria: (1) structured multidisciplinary plan of care, (2) intervention used to implement evidence into local practices, (3) intervention detailing the steps in the treatment algorithm, (4) intervention that includes criteria-based progression, and (5) intervention designed to standardize care.^13^ Clinical pathways allow for ongoing monitoring and refining of the pathway based on feedback.

Because the absolute best choice among various IOL methods has not been proven, we aimed to standardize the IOL process with a clinical pathway to improve outcomes. Our objective was to analyze whether a standardized, evidence-based IOL care pathway decreases the time from admission to delivery and improves clinical outcomes. We hypothesized that a standardized IOL care pathway would decrease the time for IOL and improve maternal and fetal outcomes.

## Materials and Methods

This was a quality improvement project to assess the effect of a standardized care pathway for IOL in a tertiary care center. The study was deemed to be a quality improvement project by the institutional review board at Naval Medical Center San Diego, San Diego, California. This institution is a large tertiary care referral center with approximately 2500 deliveries performed annually. Before the implementation of the care pathway, IOL was managed at the discretion of clinicians with potential variation in practices. This was a retrospective cohort study of patients undergoing IOL at >34 weeks of gestation between January 2018 and September 2019. IOLs were included for the 5 months before (January 2018 to May 2018) and 14 months after (August 2018 to September 2019) the implementation of the care pathway. A 2-month washout period (from June 2018 to July 2018) was performed where data were not analyzed during implementation. The primary outcome was time from admission to delivery. The secondary outcomes included chorioamnionitis, endometritis, NICU admissions, CD, PPH, and a composite of unanticipated outcomes, including chorioamnionitis, endometritis, NICU admissions, CD, and PPH.

**Figure 1:**
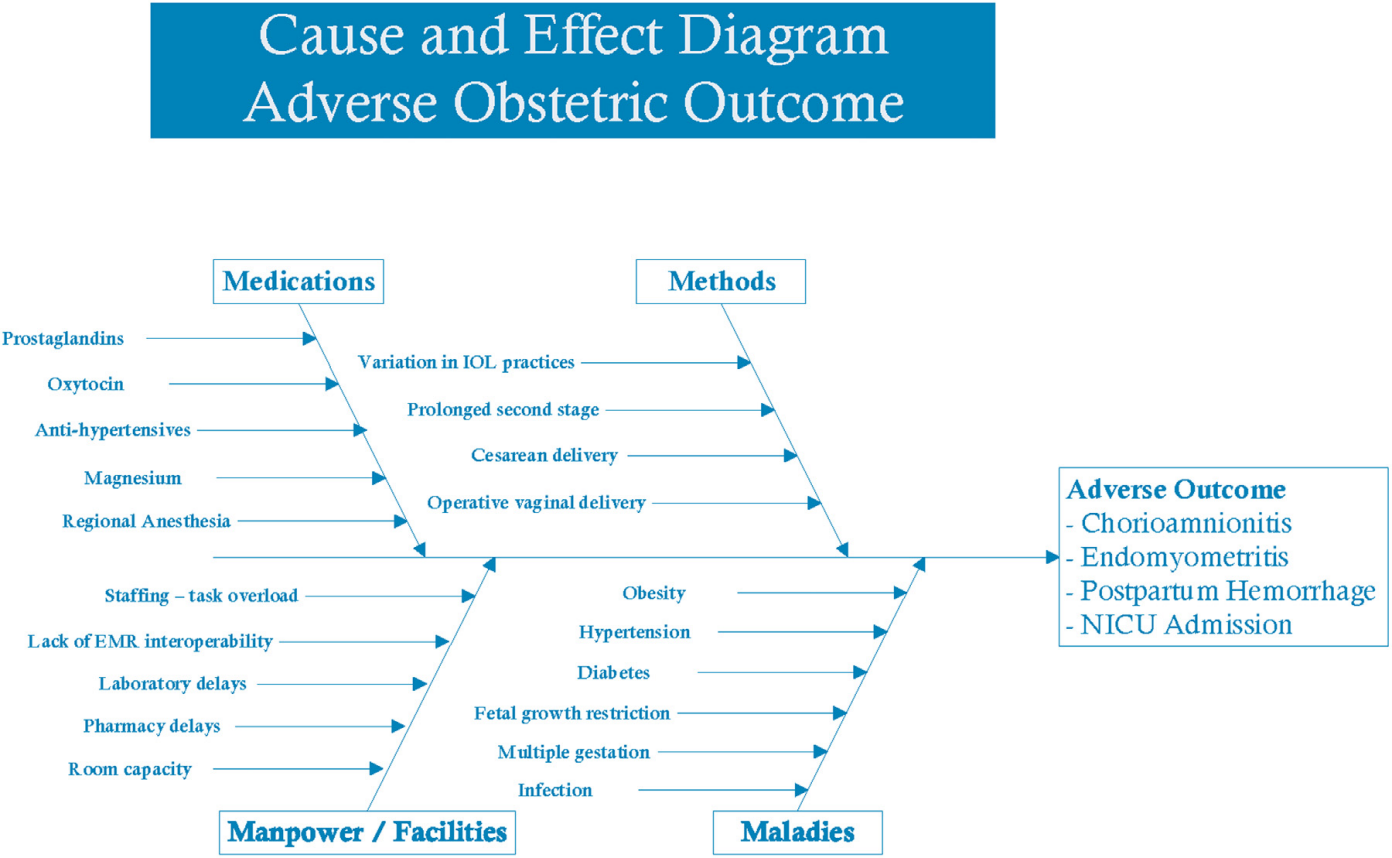
Cause and effect diagram of root causes of adverse outcomes *EMR*, electronic medical record; *NICU*, neonatal intensive care unit.

The Revised Standards for Quality Improvement Reporting guidelines^14^ and the Lean Six Sigma Define-Measure-Analyze-Improve-Control process were followed. During the define phase, a multidisciplinary team of obstetrician-gynecologists, maternal-fetal medicine physicians, anesthesiologists, neonatologists, and nurses defined the current state and made recommendations for the future state. Pathway development focused on previous studies that reported a standardized protocol for IOL.^10^^,^^11^ The team completed a cause-and-effect diagram to understand the root causes of adverse outcomes after IOL to identify drivers affecting patient outcomes (Figure 1). Multidisciplinary focus groups of clinicians and nurses identified practice gaps and solutions to improve care (Supplemental Figure 1).

Lutgendorf. Induction of labor care pathway. Am J Obstet Gynecol Glob Rep 2023.

The resulting evidence-based IOL care pathway (Supplemental Figure 2) incorporated key features:

1. Requiring gestational age of ≥39 weeks or a medical indication
2. Cervical ripening using mechanical or pharmacologic methods (misoprostol 25 μg vaginally every 4 hours or 50 μg orally every 4 hours for a max of 24 hours was preferred because of cost and effectiveness), with combination methods (misoprostol and Foley balloon catheter) preferred^15^^,^^16^
3. Recommendations for artificial rupture of the membranes within 24 hours of starting the IOL^17^^,^^18^ and placement of an intrauterine pressure catheter (IUPC) to monitor contraction strength within 6 hours of membrane rupture^11^
4. Titration of oxytocin to achieve adequate contraction Montevideo units (MVUs; 200–300)^11^
5. Use of an oxytocin checklist to assess for uterine tachysystole or fetal heart rate decelerations, with recommendations to decrease or stop oxytocin if tachysystole (>5 uterine contractions in 10 minutes for any 20-minute interval) or significant heart rate decelerations (no more than 1 late deceleration and no more than 2 variable decelerations exceeding 60 seconds in duration and decreasing >60 beats per minute from the baseline within the previous 30 minutes)^19^
6. Administration of oxytocin for at least 12 to 18 hours after membrane rupture before diagnosing a failed IOL or more than 24 hours of latent labor before diagnosing a failed IOL^6^^,^^7^
7. In active labor, diagnosis of active phase arrest if the cervix is unchanged despite adequate MVUs after ≥4 hours or inadequate contractions for ≥6 hours^6^^,^^7^
8. Arrest of descent defined as at least 2 hours of pushing in multiparous patients and at least 3 hours of pushing in nulliparous patients^6^^,^^7^

We hypothesized that this combination of interventions would decrease IOL time because of recommendations for membrane rupture and titration of oxytocin based on MVUs. Furthermore, the addition of the “Obstetric care consensus on safe prevention of primary cesarean delivery” recommendations^6^ and the addition of the oxytocin checklist^19^ were hypothesized to decrease CD rates and improve neonatal outcomes. From May 2018 to June 2018, clinicians and nurses received education on the proposed pathway and the importance of process standardization. The pathway was initiated on labor and delivery in June 2018. An IOL note was implemented in the electronic medical record (EMR) in July 2018 to document the methods of IOL and details of pathway use and deviations, and reeducation on the pathway was completed in August 2018. Planned review and revision were performed 6 months after the implementation of the care pathway (January 2019) as part of the ongoing quality improvement monitoring.

During the measure phase, outcomes were compared before and after the implementation of the care pathway. Pathway compliance was monitored using data from electronic reports, data from recorded fields, and real-time medical record reviews by trained nurse abstractors. Data were verified by study nurses to ensure accuracy and completeness. The primary outcome measure was time from admission to delivery. The secondary outcomes included infections (chorioamnionitis and endometritis), NICU admission, CD, PPH (defined as delivery with total blood loss of ≥1000 mL), a composite of one or more unanticipated outcomes (chorioamnionitis, endometritis, NICU admissions, CD, and PPH), and pathway compliance. NICU admissions were reviewed by a neonatology physician (J.O.) to ensure NICU admissions were potentially associated with IOL. NICU admissions for prematurity, congenital anomalies, and hyperbilirubinemia were not included as NICU admissions.

Compliance data were collected by tracking the following criteria: failure to active range of motion (AROM) within 24 hours of starting oxytocin, not placing an IUPC within 6 hours of ruptured membranes, diagnosing active phase arrest (arrest of dilation) at <4 hours with adequate contractions (MVUs of >200) or 6 hours with inadequate contractions after ruptured membranes, diagnosing failed IOL before 24 hours on oxytocin after cervical ripening or <12 hours on oxytocin with ruptured membranes, or arrest of descent diagnosed before 2 hours of pushing in multiparous patients or before 3 hours of pushing in nulliparous patients. If any of these criteria were met, the IOL management was considered noncompliant with the IOL care pathway.

Demographic and clinical data included age, body mass index, parity, gestational age at admission for IOL, IOL indication, and self-reported race as collected in the Defense Enrollment Eligibility Reporting System (DEERS). Racial categories used are those in DEERS, including Asian Pacific Islander, Black, White, American Indian or Alaska Native, other, and unknown.

**Figure 2:**
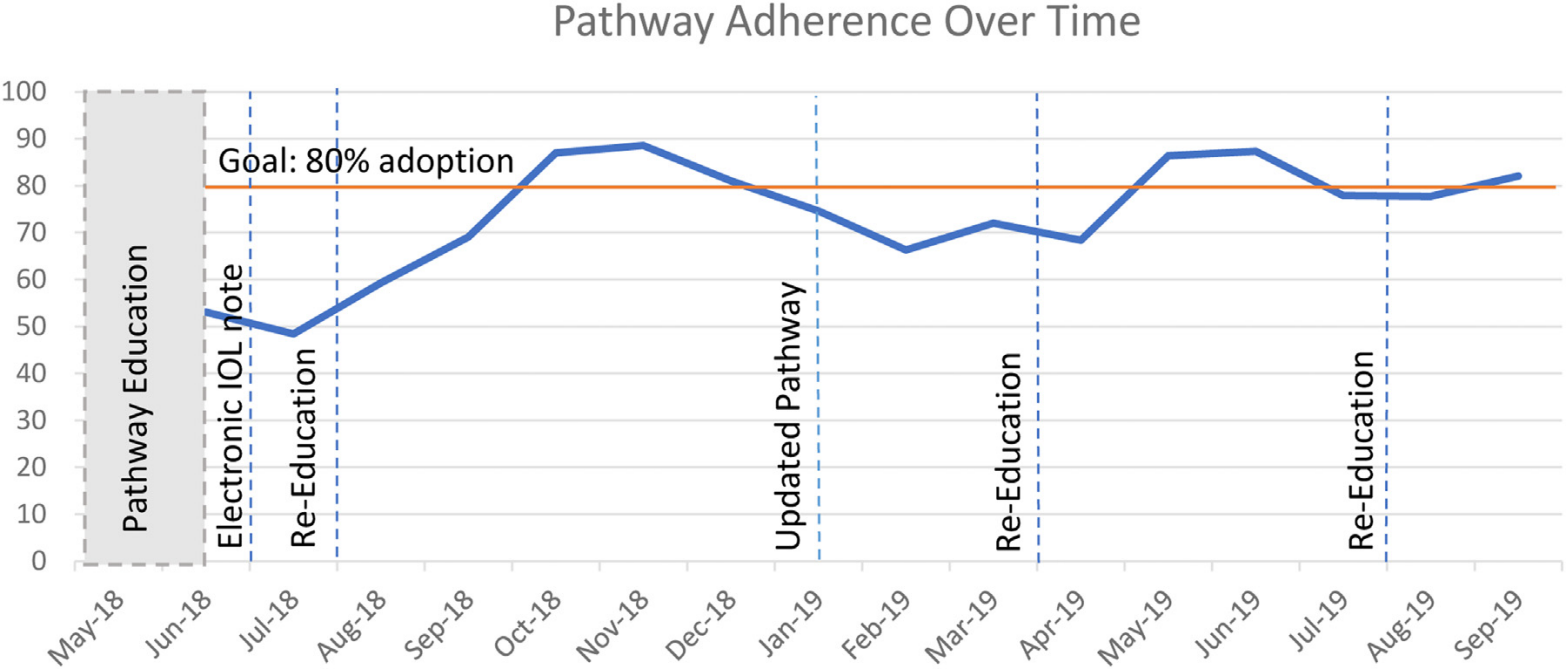
Pathway adoption over time

During the analyze, improve, and control phases, data were analyzed to determine the opportunities for improvements. Clinicians and nurses were surveyed to determine pathway use and identify necessary revisions to the pathway. Based on clinician and nurse inputs, definitions were clarified for cervical ripening and active labor using cervical examinations and the Bishop score. The second version was published in February 2019 (Supplemental Figure 2). The target compliance rate was set at 80%, anticipating that 10% to 15% of the time clinicians may need to deviate from the pathway for individual patients. Additional education was planned if significant changes to the clinical pathway or if compliance dropped below the 80% target. This was completed at 3 time points, August 2018, April 2019, and August 2019 (Figure 2). The sample size after the implementation of the care pathway needed to detect a 2-hour difference in the mean time from admission to delivery was 517, with a power of 80%, an α level of .05, and a standard deviation assumption (±12) of the observations before the pathway for both groups. A 2-hour difference from admission to delivery was selected as a clinically meaningful decrease in time. As this was an ongoing quality improvement project, data were monitored during the established period after the implementation of the care pathway to assess the outcomes and the quality improvement process.

Lutgendorf. Induction of labor care pathway. Am J Obstet Gynecol Glob Rep 2023.

Data were analyzed using a 2-tailed Student *t* test for continuous data and the chi-square or Fisher exact test for categorical data; moreover the rates of pathway compliance were calculated, along with the odds ratios (ORs) and 95% confidence interval (CIs). Statistical analysis was performed using R (version 4.1.2; R Foundation for Statistical Computing, Auckland, New Zealand). Propensity score matching was used to estimate the average marginal effect that the implementation of the care pathway had on the time from admission to delivery, accounting for confounding by covariates (age, BMI, race, ethnicity, and gestational age). Race and ethnicity are social constructs, and racial differences in outcomes represent consequences of structural racism or inequitable health opportunities based on race and ethnicity. Matching was performed using the *MatchIt* package in R. Matching with replacement was used, with a target standardized mean difference (SMD) of <0.1. SMD was used as the statistic to examine the balance of covariate distribution between groups before and after the implementation of the care pathway. As the SMD is independent of the units of measurement of the variables, it allows for comparison among variables with different units of measurement. Propensity score matching was limited to participants with complete data. To estimate each treatment effect and its standard error, a logistic regression model was fit for each outcome (hours to delivery, PPH, chorioamnionitis, endometritis, NICU admissions, CD, and composite unanticipated adverse outcomes) with predictors consisting of the treatment (protocol implementation), covariates, IOL indication, and their interaction and included the full matching weights in the estimation. The average treatment effect in the treated (ATT) was used to define the average effect of the treatment (clinical care pathway). The *glm* function was used to fit the outcome, and the *comparisons* function in the *marginaleffects* package was used to estimate the ATT.

## Results

A total of 1471 patients were included, with 392 IOLs before the implementation of the care pathway (January 2018 to May 2018) and 1079 IOLs after the implementation of the care pathway (August 2018 to September 2019). The IOL rates were 39.0% (392 IOLs among 1021 deliveries over 5 months) before the implementation of the care pathway and 40.9% (1079 IOLs among 2641 deliveries over 14 months) after the implementation of the care pathway. Maternal characteristics, IOL indications, and mode of delivery are summarized in Table 1. Patients in the postpathway group were significantly younger by 1.5 years, had more patients in the other category for self-reported race, and had a slightly more favorable Bishop score than patients in the prepathway group (Table 1). There was no statistically significant difference in IOL indications before and after the implementation of the care pathway (Table 1).

**Table 1:** Maternal characteristics and delivery mode in the pre- and postcare pathway implementation cohorts

Characteristic	Prepathway cohort: Jan. 2018 to May 2018 (n=392)	Postpathway cohort: Aug. 2018 to Sept. 2019 (n=1079)	*P* value	Prematch SMD (n=299)	Postmatch SMD (n=1070)
IOL rate, n/N (%)	392/1021 (39.0)	1079/2641 (40.9)			
Age (y)	31.0±5.3	29.4±5.3	<.001	−0.260	−0.022
BMI (kg/m^2^)	32.5±6.0	32.6±5.9	.71	−0.008	0.056
Race, n (%)					
American Indian or Alaska Native	1 (0.3)	8 (0.7)	—	0.050	−0.061
Asian Pacific Islander	25 (6.4)	59 (5.5)	.036	−0.033	−0.005
Black	56 (14.3)	168 (15.6)		0.030	0.017
Other	91 (23.2)	322 (29.8)		0.119	0.09
Unknown	10 (2.6)	13 (1.2)		−0.160	0.074
White	209 (53.3)	509 (47.2)		−0.089	−0.099
Ethnicity, n (%)					
Hispanic	45 (11.5)	184 (17.1)	.009	0.119	0.025
non-Hispanic	347 (88.5)	895 (82.9)			
Parity					
Nulliparous	244 (60.7)	702 (65.1)	.128	−0.061	0.028
Multiparous	158 (39.3)	377 (34.9)			
Bishop score (mean)	3.16	4.05	<.001	0.372	0.049
Gestational age (wk)	38 5/7	38 6/7	.43	0.029	0.043
<36 0/7	20 (5.1)	45 (4.1)	.79		
36 0/7 to 38 6/7	145 (37.0)	426 (39.5)			
39 0/7 to 39 6/7	91 (23.2)	259 (24.0)			
40 0/7 to 40 6/7	88 (22.4)	221 (20.5)			
≥41 0/7	48 (12.2)	127 (11.8)			
Mode of delivery					
Vaginal	289 (73.7)	823 (76.2)	.56	—	—
Operative vaginal	17 (4.3)	35 (3.2)		—	—
Cesarean	77 (19.6)	217 (20.1)		—	—
IOL indication					
FHR abnormality	32 (8.2)	50 (4.6)	.09	−0.201	0.048
Hypertensive disorders	134 (34.1)	429 (39.8)		0.133	−0.040
Fetal growth restriction	7 (1.8)	22 (2.0)		−0.019	0.00
Fetal indications	4 (1.0)	6 (0.6)		−0.075	0.013
Elective≥39 wk	48 (12.2)	162 (15.0)		0.072	−0.049
Postdates≥41 wk	56 (14.3)	127 (11.8)		−0.098	0.066
Diabetes mellitus	14 (3.6)	44 (4.0)		0.036	−0.016
Intrahepatic cholestasis	16 (4.1)	45 (4.1)		0.069	−0.005
Oligohydramnios	8 (2.0)	29 (2.7)		0.073	0.00
Other	42 (10.7)	84 (7.8)		−0.119	−0.035
Ruptured membranes	29 (7.4)	80 (7.4)		−0.040	0.068

Some columns may not add to 100% because of missing data. Chi-square analysis was used to calculate for age, race, gestational age categories, delivery method, and IOL indication. The Fisher exact test was used to calculate for ethnicity. The 2 sample *t* test was used to calculate for age, mean gestational age, and BMI.

*BMI*, body mass index; *FHR*, fetal heart rate; *IOL*, induction of labor; *SMD*, standardized mean difference.

After the implementation of the IOL care pathway, there was a nonsignificant reduction in the time from admission to delivery by 1.2 hours (from 23.4 to 22.1 hours; *P*=.08) (Table 2). When stratified by mode of delivery, there was a nonsignificant increase in the time to CD before (28.2 hours) and after (28.8 hours) protocol implementation (*P*=.71). For vaginal deliveries, there was a significant decrease in the time to delivery by 1.7 hours after protocol implementation (from 22.2 to 20.5 hours; *P*=.02). For the secondary outcomes, there was a significant decrease in chorioamnionitis by 6.5% (from 12.5% to 6.0%; OR, 0.44; 95% CI, 0.29–0.67), a significant decrease in endometritis by 4.3% (from 6.9% to 2.6%; OR, 0.36; 95% CI, 0.20–0.65), and a significant decrease in the composite unanticipated outcomes by 20.3% (from 56.9% to 36.6%; OR, 0.44; 95% CI, 0.34–0.56). There was no significant difference in NICU admissions (from 18.1% to 14.0%; OR, 0.74; 95% CI, 0.54–1.02), CDs (from 19.6% to 20.1%; OR, 1.03; 95% CI, 0.76–1.40), or PPH (from 7.9% to 6.1%; OR, 0.76; 95% CI, 0.48–1.22). Of the IOLs that occurred after the implementation of the care pathway, 897 (75%) were adherent to the pathway. Pathway adherence ranged between 50% and 89% throughout the implementation process as shown in Figure 2, with pathway education and reeducation time points noted. When compliance was stratified into IOL methods (failure to AROM within 24 hours of starting oxytocin and not placing an IUPC within 6 hours of ruptured membranes) compared with CD indications (diagnosing active phase arrest at <12 to 18 hours on oxytocin with ruptured membranes, diagnosing failed IOL <24 hours on oxytocin, diagnosing arrest of dilation <4 hours of adequate contractions after membrane rupture or <6 hours of inadequate contractions, and arrest of descent before 2 hours of pushing in multiparous patients and before 3 hours of pushing in nulliparous patients), 165 cases (69%) were attributed to deviations from IOL methods, and 74 cases (31%) were deviations from CD indications.

**Table 2:** Comparison of clinical outcomes for IOL before and after the implementation of an IOL care pathway

Outcome metric	Prepathway implementation: Jan. 2018 to May 2018 (n=392)	Postpathway implementation: Aug. 2018 to Sept. 2019 (n=1079)	OR (95% CI)	*P* value	Completed match ATT^a^	Completed match *P* value^a^
Time from admission to delivery (h)	23.4±12.0	22.2±11.2	—	.08	−1.200	.026
Chorioamnionitis	49 (12.5)	64 (6.0)	0.44 (0.29–0.67)	<.001	−0.046	.003
Endometritis	27 (6.9)	28 (2.6)	0.36 (0.20–0.65)	<.001	−0.051	.001
PPH	31 (7.9)	66 (6.1)	0.76 (0.48–1.22)	.23	−0.098	.413
NICU admissions	71 (18.1)	177 (14.0)	0.74 (0.54–1.02)	.058	−0.017	.419
CDs^b^	77 (19.6)	217 (20.1)	1.03 (0.76–1.40)	.88	−0.014	.51
Unanticipated outcomes associated with IOL (chorioamnionitis, endometritis, NICU admissions, CD, and PPH)	223 (56.9)	395 (36.6)	0.44 (0.34–0.56)	<.001	−0.215	<.001

Data are presented as mean±SD or number (percentage), unless otherwise indicated. Some columns may not add to 100% because of missing data.

*ATT*, average treatment effect on the treated; *CD*, cesarean delivery; *CI*, confidence interval; *IOL*, induction of labor; *NICU*, neonatal intensive care unit; *OR*, odds ratio; *PPH*, postpartum hemorrhage; *SD*, standard deviation.

a Generated from propensity score matching with replacement, including only patients with complete variables (n=1491);

b Delivery method not available for 9 predeliveries and 4 postdeliveries.

When stratified by parity, nulliparous patients had a nonsignificant 1.5-hour decrease in the time to delivery, from 26.5 before protocol implementation to 25 hours after protocol implementation (*P*=.09). Furthermore, nulliparous patients had a significantly shorter time from admission to vaginal delivery by 2.5 hours after protocol implementation, from 25.5 hours before protocol implementation to 23 hours after protocol implementation (*P*=.01), and there was a nonsignificant increase in the time to CD for nulliparous patients, from 29.3 hours before protocol implementation to 30.9 hours after protocol implementation (*P*=.42). All delivery times were shorter for multiparous patients than nulliparous patients, with nonsignificant decreases in time to delivery from 18.2 hours before protocol implementation to 17 hours after protocol implementation (*P*=.17). There was a nonsignificant decrease in the time to vaginal delivery for multiparous patients from 17.9 hours before protocol implementation to 16.5 hours after protocol implementation (*P*=.14), and there was a nonsignificant decrease in the time to CD, from 21.8 hours before protocol implementation to 20.4 hours after protocol implementation (*P*=.68). In addition, multiparous patients had significantly lower rates of protocol deviations (14.3%) than nulliparous patients (26.4%) (*P*<.001).

Propensity score matching was used to estimate the average marginal effect of the IOL care pathway on the time from admission to delivery (SMD) compared with prepathway deliveries. The optimal matching estimate yielded adequate balance as indicated in Table 1, and covariate balance is displayed in the Love plot shown in Supplemental Figure 3. A total of 161 records were excluded for missing data (52 in the prepathway group and 103 in the postpathway group). Of the remaining 334 individuals in the prepathway group, 82 were discarded with matching, leaving 252 individuals in the prepathway group and 976 individuals in the postpathway group for propensity score matching. Based on the matching, the estimated ATT of the implementation of the care pathway included a significantly lower risk of composite unanticipated outcomes, chorioamnionitis, endometritis, and time from admission to delivery. The rates of CD, NICU admission, and PPH had nonsignificant decreases, as shown in Table 2.

## Comment

### Principal findings

The implementation of a standardized IOL care pathway was associated with a small nonsignificantly shorter time from admission to delivery of 1.2 hours and improved patient outcomes, including decreased risks of chorioamnionitis, endometritis, and composite unanticipated outcomes. This emphasizes that, even in the absence of large randomized controlled clinical trials, implementing an evidence-based practice and decreasing clinical variation are associated with improved outcomes.

### Results

Similar to previous studies, we noted small improvements in the time to delivery with a standardized IOL protocol.^11^^,^^12^ These improvements in the time to delivery were primarily driven by a significant decrease in the time to vaginal delivery by 1.7 hours from 22.2 hours before protocol implementation to 20.5 hours after protocol implementation (*P*=.02). Nulliparous patients had a significantly shorter time to vaginal delivery by 2.5 hours after protocol implementation; thus, this intervention may be particularly effective in decreasing the time to delivery in nulliparous patients. Our study demonstrated improvements in infections and NICU admissions, which were not reported previously.^11^^,^^12^

### Clinical implications

The data suggest that a care pathway, including standardized management of IOL, is associated with improved outcomes and could be implemented in other practices. Improvements in obstetrical outcomes have been reported for standardized management of hypertension, with decreases in maternal mortality from the leading cause of maternal death to the 11th cause of maternal death.^20^ Standardized oxytocin in-use checklists decrease oxytocin infusion rates, decrease CDs, and improve neonatal outcomes.^19^^,^^20^ Oxytocin checklists were a part of our IOL care pathway and may have contributed to improved outcomes. Before the implementation of the IOL care pathway, oxytocin checklists were used by nursing staff to document the initiation of oxytocin and for assessments in labor^21^; however, there was no protocol to decrease or stop oxytocin based on checklist results, and management was at the discretion of individual clinicians.

Care pathways are standardized approaches to care that improve outcomes by decreasing clinical variation. This care pathway was based on published protocols emphasizing cervical ripening, early amniotomy with IUPC placement to titrate oxytocin, and standardized definitions for the failure of IOL.^10^^,^^11^ Combination methods, including a Foley catheter and misoprostol^15^^,^^22^ or oxytocin,^16^ were recommended because of decreased time to delivery, with a recent meta-review demonstrating that a combination of a single-balloon catheter and misoprostol was the most effective approach to IOL with increased odds of vaginal delivery within 24 hours, decreased CD, and decreased NICU admissions.^22^

Although clinicians were concerned about potential increased infections with membrane rupture and IUPC placement, significant decreases in chorioamnionitis and endometritis rates occurred after the implementation of the care pathway. These results are consistent with other studies that did not demonstrate increased infection rates with early amniotomy and placement of an IUPC for monitoring IOL.^11^^,^^23^ It is possible that IUPC use improved the ability to accurately monitor contractions and optimize titration of oxytocin, resulting in a significantly shorter IOL time, decreased uterine tachysystole, and improved outcomes. Furthermore, although placement of an IUPC is not required for labor management or to diagnose labor arrest disorders,^7^ palpation of the strength of uterine contractions requires additional hands-on assessments and may not be regularly applied in clinical practice. Moreover, active management of IOL with early amniotomy has been shown in nulliparous patients to lead to shorter labor with less labor dystocia, lower CD rates, lower abruption rates, and no increase in complications.^17^^,^^18^

### Research implications

The care pathway was developed using quality improvement assessment practices to assess potential drivers of adverse outcomes related to IOL and was based on the best available evidence. Wider adoption of standardized IOL protocols at multiple institutions would be beneficial to assess the effect of more widespread standardization.

### Strengths and limitations

The strengths of this study include the relatively large cohort of patients undergoing induction over a 15-month period. The involvement of a multidisciplinary team in protocol development and education and increased compliance over time were other strengths. The care pathway also included a focus on potential drivers of adverse outcomes and was designed to improve outcomes and decrease the time to delivery. Another strength of this study includes data extraction by nurse abstractors, which improved data quality, as they were able to validate compliance data.

The study also had limitations. Despite education, pathway compliance variably ranged between 50% and 89%. This is common with new protocols, and compliance improved with reeducation (Figure 2). Another limitation was that IOL time was calculated from the time of admission in the EMR, and there may have been variation in the timing of interventions. This was a nonrandomized study, with possible selection bias. However, prepathway and postpathway groups had similar baseline characteristics and prognoses, and similar results were achieved with propensity score matching. Although large randomized controlled trials have not clarified the optimal approach to IOL,^8^ the standardized protocol included the best available evidence,^6^^,^^7^^,^^9^^,^^17^, ^18^, ^19^, ^20^, ^21^^,^^23^ experience from observational trials,^10^^,^^11^ and a recent meta-review.^22^ The care pathway recommended amniotomy within 24 hours of starting oxytocin; however, an earlier amniotomy within 1 to 4 hours after cervical ripening^12^^,^^24^ may have affected the study outcomes. Finally, the decreased time to delivery was relatively small and nonsignificant, 1.2 hours less than before the implementation of the care pathway, and this may not be considered clinically meaningful.

As this was an observational study, we were unable to draw conclusions regarding causality, and although we standardized important aspects of IOL, other potential variations in practices may remain and may change over time. During the study period, there was no other significant institutional practice change, such as blood loss assessment, diagnostic criteria for chorioamnionitis or endometritis, and criteria for NICU admission. Our study was also limited by the relatively small amount of preprotocol data that did not allow for an interrupted time series analysis, and we cannot control for any potential trends in the data. However, a visual review of the graphed data of time to delivery did not demonstrate any significant or sustained trends. We also cannot mitigate the potential effect of the Hawthorne effect, where individuals may have changed behavior because of ongoing evaluation. However, the implementation of a care pathway is a generalizable real-world practice that could be implemented at other institutions.

### Conclusions

A quality improvement initiative with the implementation of a standardized IOL care pathway was associated with improved patient outcomes, including a small, nonsignificantly decreased time from admission to delivery (1.2 hours), decreased infections, and decreased unanticipated outcomes. Similar process standardizations for IOL in other practice settings may similarly decrease clinical variation and improve outcomes.

## Appendix Supplementary materials

Image, application 1

Image, application 2

Image, image 3

## Footnotes

Supplementary material associated with this article can be found in the online version at doi:10.1016/j.xagr.2023.100292.

## References

[1] Simpson KR (2022). Trends in labor induction in the United States, 1989 to 2020. MCN Am J Matern Child Nurs.

[2] Grobman WA, Rice MM, Reddy UM (2018). Labor Induction versus Expectant Management in Low-Risk Nulliparous Women. N Engl J Med.

[3] Hong J, Atkinson J, Roddy Mitchell A (2023). Comparison of Maternal Labor-Related Complications and Neonatal Outcomes Following Elective Induction of Labor at 39 weeks of Gestation vs. expectant Management. JAMA Netw Open.

[4] (2020). Gestational hypertension and preeclampsia: ACOG practice bulletin, number 222. Obstet Gynecol.

[5] American College of Obstetricians and Gynecologists' Committee on Practice Bulletins—Obstetrics (2019). ACOG Practice Bulletin No. 203: chronic hypertension in pregnancy. Obstet Gynecol.

[6] (2014). Safe prevention of the primary cesarean delivery. Obstetric care Consensus No. 1. American College of Obstetricians and Gynecologists. Obstet Gynecol.

[7] (2009). ACOG Practice Bulletin No. 107: induction of labor. Obstet Gynecol.

[8] Ayala NK, Rouse DJ (2019). Nondefinitive studies of labor induction methods: enough already!. Obstet Gynecol.

[9] Clark SL, Belfort MA, Byrum SL, Meyers JA, Perlin JB (2008). Improved outcomes, fewer cesarean deliveries, and reduced litigation: results of a new paradigm in patient safety. Am J Obstet Gynecol.

[10] Rouse DJ, Owen J, Hauth JC (2000). Criteria for failed labor induction: prospective evaluation of a standardized protocol. Obstet Gynecol.

[11] Rhinehart-Ventura J, Eppes C, Sangi-Haghpeykar H, Davidson C (2014). Evaluation of outcomes after implementation of an induction-of-labor protocol. Am J Obstet Gynecol.

[12] Suresh SC, Kucirka L, Chau DB, Hadley M, Sheffield JS (2020). Evidence-based protocol decreases time to vaginal delivery in elective inductions. Am J Obstet Gynecol MFM.

[13] Kinsman L, Rotter T, James E, Snow P, Willis J (2010). What is a clinical pathway? Development of a definition to inform the debate. BMC Med.

[14] Ogrinc G, Davies L, Goodman D, Batalden P, Davidoff F, Stevens D (2016). SQUIRE 2.0 (Standards for Quality Improvement Reporting Excellence): revised publication guidelines from a detailed consensus process. BMJ Qual Saf.

[15] Ornat L, Alonso-Ventura V, Bueno-Notivol J, Chedraui P, Pérez-López FR, Health Outcomes and Systematic Analyses (HOUSSAY) Research Group (2020). Misoprostol combined with cervical single or double balloon catheters versus misoprostol alone for labor induction of singleton pregnancies: a meta-analysis of randomized trials. J Matern Fetal Neonatal Med.

[16] Gallagher LT, Gardner B, Rahman M (2019). Cervical ripening using foley balloon with or without oxytocin: a systematic review and meta-analysis. Am J Perinatol.

[17] Kim SW, Nasioudis D, Levine LD (2019). Role of early amniotomy with induced labor: a systematic review of literature and meta-analysis. Am J Obstet Gynecol MFM.

[18] Macones GA, Cahill A, Stamilio DM, Odibo AO (2012). The efficacy of early amniotomy in nulliparous labor induction: a randomized controlled trial. Am J Obstet Gynecol.

[19] Clark S, Belfort M, Saade G (2007). Implementation of a conservative checklist-based protocol for oxytocin administration: maternal and newborn outcomes. Am J Obstet Gynecol.

[20] Clark SL, Christmas JT, Frye DR, Meyers JA, Perlin JB (2014). Maternal mortality in the United States: predictability and the impact of protocols on fatal postcesarean pulmonary embolism and hypertension-related intracranial hemorrhage. Am J Obstet Gynecol.

[21] Institute for Healthcare Improvement. The oxytocin bundle. Available at:http://www.ihi.org/education/WebTraining/Expeditions/Oxytocin/Pages/default.aspx. Accessed January 28, 2023.

[22] Sanchez-Ramos L, Lin L, Vilchez-Lagos G, et al. Single-balloon catheter with concomitant vaginal misoprostol is the most effective strategy for labor induction: a meta-review with network meta-analysis. Am J Obstet Gynecol [in press]. 10.1016/j.ajog.2022.01.005 38462253

[23] Bakker JJ, Janssen PF, van Halem K (2013). Internal versus external tocodynamometry during induced or augmented labour. Cochrane Database Syst Rev.

[24] Gomez Slagle HB, Fonge YN, Caplan R, Pfeuti CK, Sciscione AC, Hoffman MK (2022). Early vs expectant artificial rupture of membranes following Foley catheter ripening: a randomized controlled trial. Am J Obstet Gynecol.

