# Supplementary material for: Pregnancy outcomes after implementation of an induction of labor care pathway
Source: AJOG Glob Rep. 2023 Nov 18;4(1):100292. doi: 10.1016/j.xagr.2023.100292 (PMC10750180; doi:10.1016/j.xagr.2023.100292)
Supplement: Supplementary file 1 [file mmc1.docx]

Supplemental Figure 1. Countermeasures developed to address practice gaps

| **Gap(s) Addressed** | **Solution** | **Expected Outcome** |
| --- | --- | --- |
| Variation in IOL practices | Evidence based clinical practice | Decrease non-beneficial clinical variation |
| - Inconsistent cervical ripening | Adequate time for ripening | Decrease cesarean deliveries |
| - Inconsistent definitions of failed IOL | Clearly define failed IOL | Prevent inconsistent definitions and non-indicated cesarean deliveries |
| - Inconsistent definitions of active phase arrest (arrest of dilatation) | Define active phase arrest (arrest of dilatation) | Prevent inconsistent definitions and non-indicated cesarean deliveries |
| - Inconsistent AROM and IUPC use | AROM and IUPC recommendations | Decrease time in labor and decrease infections and postpartum hemorrhage |
| - Oxytocin administration variable | Define criteria to stop or decrease oxytocin infusion | Decrease cesarean deliveries for non-reassuring fetal status, improve neonatal outcome |
| Prolonged second stage | Clear definitions for prolonged second stage | Decrease non-indicated cesarean deliveries |
| Staffing shortages / Task overload | Clear clinical algorithm | Decrease non-beneficial clinical variation |
| Room capacity | Decreased time in induction | Increase capacity |
