# Supplementary material for: Pregnancy outcomes after implementation of an induction of labor care pathway
Source: AJOG Glob Rep. 2023 Nov 18;4(1):100292. doi: 10.1016/j.xagr.2023.100292 (PMC10750180; doi:10.1016/j.xagr.2023.100292)
Supplement: Supplementary file 2 [file mmc2.pdf]

Perinatal Care Pathway: Induction of Labor

Management of Induction of Labor When the Fetal Heart Rate is Reassuring

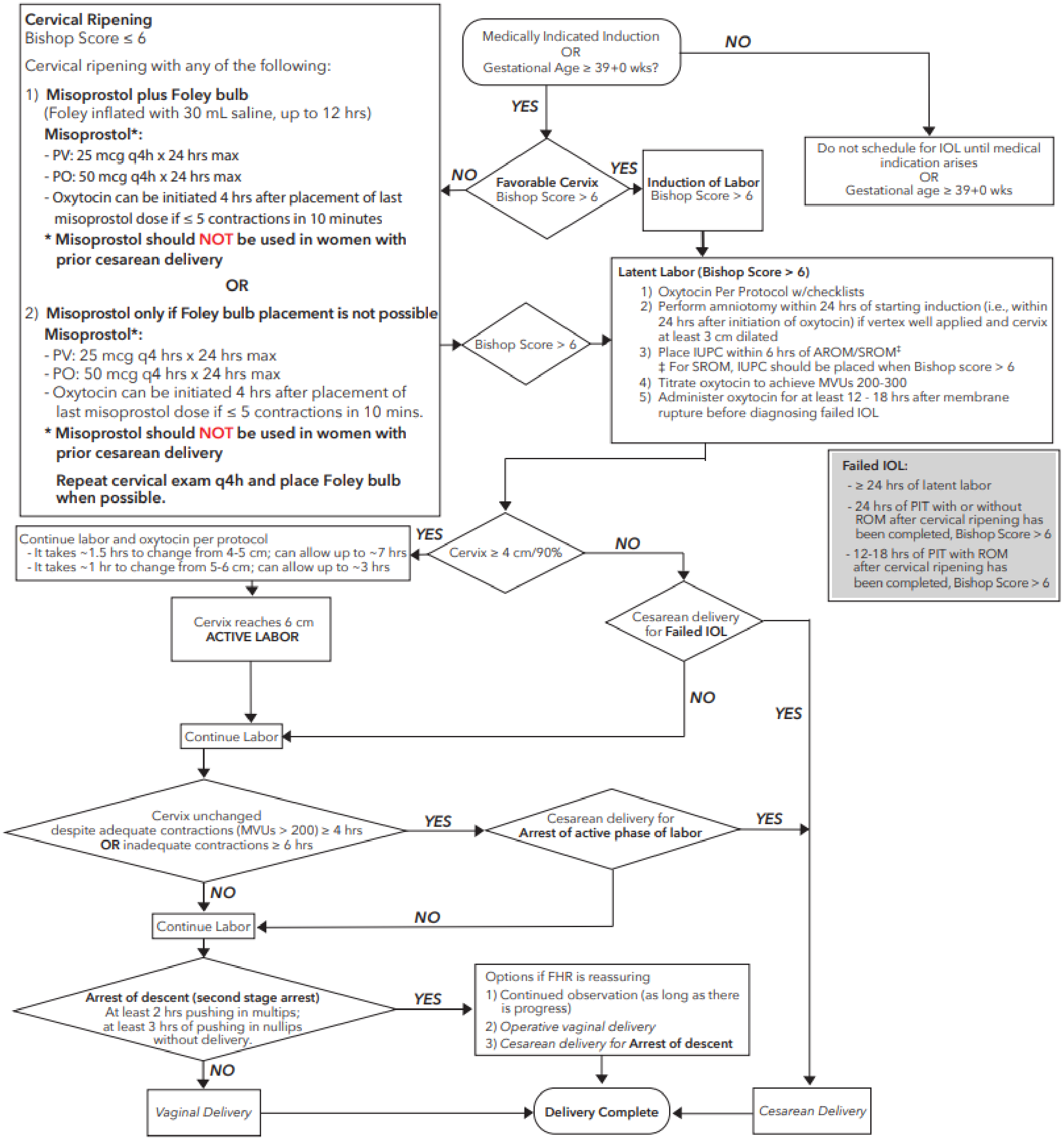

Perinatal Care Pathway: Induction of Labor

Pre PIT (oxytocin) checklist*

If the following checklist cannot be completed, oxytocin should not be initiated.

[ ] Minimum of 30 minutes monitoring

[ ] At least 2 accelerations (15 bpm x 15 seconds) in 30 minutes are present, or a biophysical profile of 8 out of 10 is present within the past 4 hrs, or moderate FHR variability.**

[ ] No late decelerations in the last 30 minutes.

[ ] No more than 2 variable decelerations exceeding 60 seconds and decreasing > 60 bpm from baseline within the previous 30 minutes prior to starting PIT infusion.

PIT administration and documentation*

During PIT administration, the following will be documented every 30 minutes. Oxytocin should be stopped or decreased if the following checklist cannot be completed.

✓FETAL ASSESSMENT

[ ] At least 1 acceleration (15 bpm x 15 seconds) in 30 minutes, or moderate variability for 10 of the previous 30 minutes

[ ] No more than 1 late deceleration in the last 30 minutes

[ ] No more than 2 variable decelerations exceeding 60 seconds and decreasing > 60 bpm from baseline within the previous 30 minutes

✓UTERINE CONTRACTIONS

[ ] No more than 5 uterine contractions in 10 minutes for any 20-minute interval

[ ] No two contractions > 120 seconds in duration

[ ] Uterus palpates soft between contractions

[ ] If IUPC in place, MVU (Montevideo units) must calculate < 300 mmHg, and baseline resting tone < 25 mmHg

* **If oxytocin is stopped, the Pre PIT checklist will be completed before oxytocin is reinitiated.**

** There will be some situations in which alterations in the management from that described in the protocol are clinically appropriate. If, after reviewing the fetal heart rate strip and course of labor, the responsible staff provider feels that, in his or her judgement, continued use of PIT is in the best interest of the mother and baby, the staff provider should write a note to that effect and order the PIT to continue. The RN will continue to provide safe, high-quality nursing care.

References

Clark S, Belfort M, Saade G, et al. Implementation of a conservative checklist-based protocol for oxytocin administration: maternal and newborn outcomes. Am J Obstet Gynecol. 2007;480.e1-480.e5.

Rhinehart-Ventura J, Eppes C, Sangi-Haghpeykar H, Davidson C. Evaluation of outcomes after implementation of an induction-of-labor protocol. Am J Obstet Gynecol. 2014;211(3):301.e1-e7.

Definitions and notes

(a) Failed induction of labor (IOL):

- ≥ 24 hrs of latent labor.
- 24 hrs of PIT with or without ROM after cervical ripening has been completed.
- 12-18 hrs of PIT with ROM.

End of latent labor: 6 cm dilation.

(b) Arrest of dilation in first stage of labor (≥ 6 cm dilation)/active phase arrest: ≥ 4 hrs of adequate contractions after AROM OR ≥ 6 hrs of inadequate contractions after AROM and no cervical change.

(c) Arrest of labor in second stage (after complete dilation)/arrest of descent: At least 2 hrs pushing in multips; at least 3 hrs of pushing in nullips.

(d) Augmentation: Cervix ≥ 5-6 cm with PIT initiated for labor protraction (≥ 2 hrs with inadequate uterine activity) or labor arrest.

(e) Medically indicated: Complication or condition that results in recommendation for induction.

(f) Adequate PIT MVUs: ≥200 MVU (1 ctx Q 2-3 minutes, lasting 80-90 seconds).

(g) AROM: AROM within 24 hrs of starting PIT (after cervical ripening).

(h) IUPC: Place within 6 hrs of AROM.

Low-dose Pitocin (PIT) (oxytocin) protocol

i. Begin PIT infusion at 2 mUnits/minute.

ii. Increase by 2 mUnits/minute every 30 minutes until an adequate contraction pattern is achieved and meeting "PIT Assess" criteria in the "Maternal Ante/ Intrapartum" flowscreen.

High-dose Pitocin (PIT) (oxytocin) protocol

Note: Contraindicated in Trial of Labor After Cesarean (TOLAC) and history of uterine surgery.

i. Begin PIT at 4mUnits/minute.

ii. Increase by 4 mUnits/minute every 30 minutes until an adequate contraction pattern is achieved meeting "PIT Assess" criteria in the "Maternal Ante/ Intrapartum" flowsheet.

Pitocin (oxytocin) notes

- 1⁄2 life = 5 minutes; steady-state = 40 minutes.
- Pitocin should not be administered < 4 hrs from last MISO dose.
- Dose greater than 20 mUnits/minute requires a provider order.
