# Supplementary figures and images for: Pregnancy outcomes after implementation of an induction of labor care pathway
Source: AJOG Glob Rep. 2023 Nov 18;4(1):100292. doi: 10.1016/j.xagr.2023.100292 (PMC10750180; doi:10.1016/j.xagr.2023.100292)

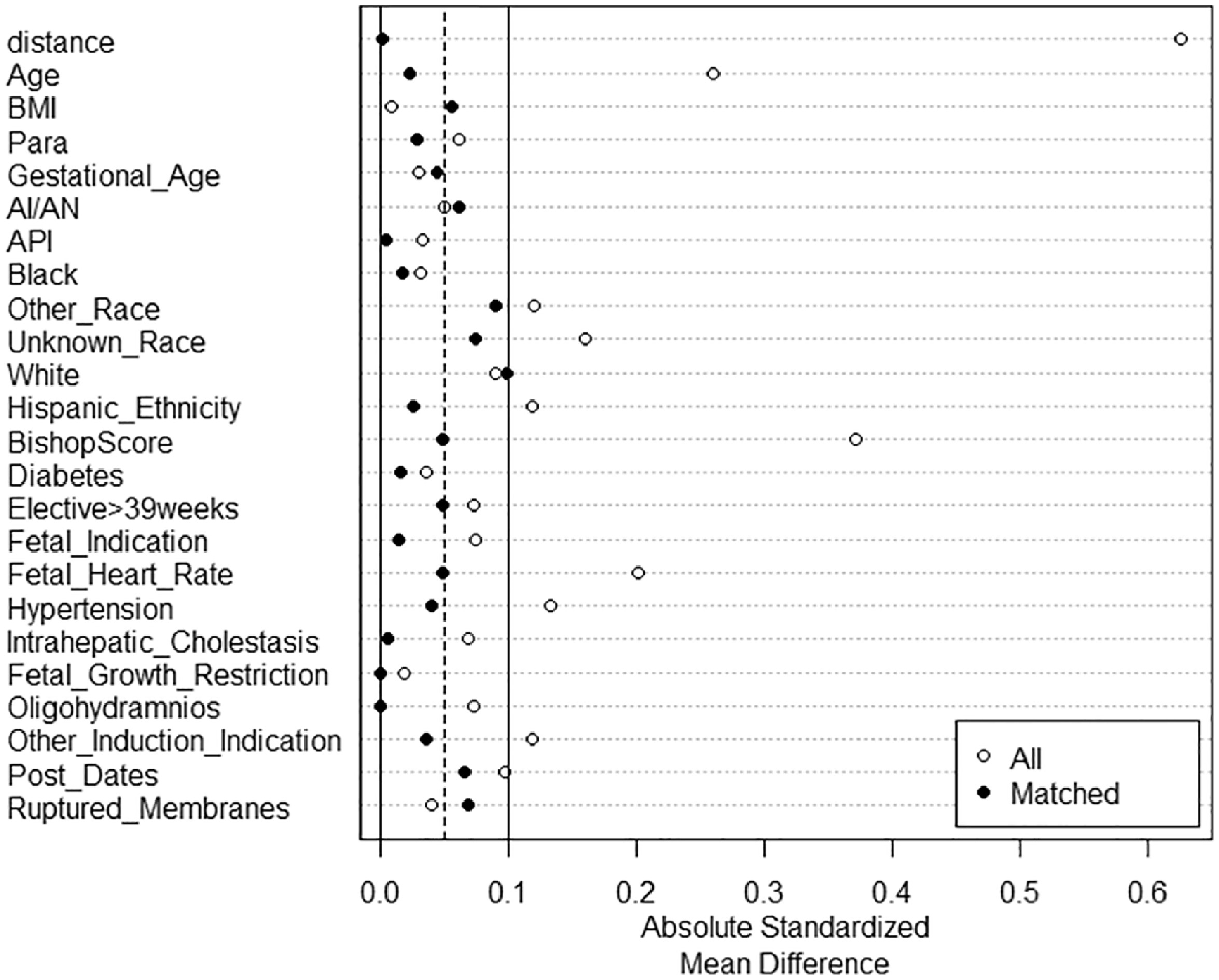

Supplement: Supplementary file 3 [file mmc3.jpg]
